# Entangled Bonds: Dyadic Dependence and Co-Regulation in Western Urban Human–Dog Relationships

**DOI:** 10.3390/ani16050715

**Published:** 2026-02-25

**Authors:** Agnieszka Grynkiewicz, Anna Reinholz, Kamil Imbir

**Affiliations:** Department of Psychology, University of Warsaw, 02-097 Warsaw, Poland; areinholz@psych.uw.edu.pl (A.R.); kimbir@psych.uw.edu.pl (K.I.)

**Keywords:** emotional co-regulation, physiological synchrony, social buffering, human–dog interactions, attachment, affective dependence, overprotection, embodied regulation, interspecific synchronization, urban social life

## Abstract

Urban contemporary living has increasingly shifted emotional regulation inward, away from wider social networks and into tightly managed daily life. Within this landscape, dogs can become regulatory partners whose presence reshapes human rhythms, attention, and everyday sociability. This review examines how urban conditions—including risk-averse caregiving, dense living, and reduced opportunities for sustained social contact—reconfigure emotional co-regulation within human–dog relationships and, in turn, human emotional environments. Drawing on research from behavioural science, physiology, comparative ethology, psychology, and the social sciences (2010–2025), it treats attachment, synchrony, and social buffering as interconnected processes. Across disciplines, evidence suggests that dogs and humans often settle emotionally together, showing coupled dynamics in behaviour and physiology. Such coupling can support stress buffering and recovery, yet under chronic human stress or heightened control it may stabilise shared vigilance and dependence, concentrating regulatory work within the dyad. These effects are conditional: when dogs lack stable, reciprocal social buffering—especially with conspecifics—the dyad may be less able to support recovery, and synchrony may tilt toward vigilance rather than calm. Seen this way, human–dog bonds function as part of the emotional infrastructure of contemporary cities, shaping how calm, uncertainty, and social contact are organised.

## Simple Summary

City life is increasingly optimised and predictable, yet often less physically shared. In this context, dogs do more than comfort people. By requiring daily walks and shared routines, they pull humans back into the physical world—weather, movement, waiting, detours—and this “analog friction” can support everyday emotional recovery. Dogs also change how people meet in public space. Their presence lowers the barrier for brief, low-stakes interaction, supports repeated recognition, and can help build weak but lasting neighbourhood ties. This review brings together research from behavioural science, physiology, psychology, and social sciences to show that humans and dogs often regulate emotions together. This coupling can buffer stress, but it is not always beneficial. Under chronic stress or heightened control, the same closeness may stabilise shared vigilance and dependence, especially when dogs themselves lack access to stable social buffering (including contact with other dogs). Seen this way, human–dog relationships are part of the emotional and social infrastructure of contemporary cities, with benefits for humans that may carry costs for dogs.

## 1. Introduction

Urban social life increasingly concentrates emotional regulation within close relationships, and dogs have become key partners through whom everyday calm, reassurance, and stability are organised. Urban human life offers frequent interaction, yet often lacks continuity [1,2]. Contacts multiply, while relationships rarely have time to settle. At the same time, a growing share of interaction has moved from physically shared settings into digitally mediated spaces, preserving contact while reducing embodied co-presence and everyday opportunities for emotional regulation [3,4,5,6].

A similar pattern now shapes canine experience [7]. Dogs encounter many others but seldom form stable social bonds. The compression of human social networks—driven by digital mediation and heightened sensitivity to risk—echoes in their lives as well [4,7,8]. Care becomes increasingly organised through supervision, and affection is expressed through management, reflecting broader cultural shifts in caregiving shaped by moralised responsibility and risk avoidance [9,10]. Beneath this, both species may adapt to a shared state of vigilance, in which calm is maintained less through recovery than through restraint [11,12,13].

This article explores that convergence. It examines how modern guardianship, shaped by concern for safety and moralised responsibility, reshapes the emotional architecture of the human–dog bond, giving rise to what may be described as controlled affection: a form of care that protects by limiting, connects by supervising, and soothes by constraining uncertainty [1,7,9]. Alongside attachment and care norms, we treat dog–human coordination (behavioural and physiological) [14,15] as a pathway through which everyday rhythms, attention [16,17,18], and low-stakes sociability in public space [3,19] can be shaped.

Importantly, this shift extends beyond the human–dog pair. As dogs take on functions once distributed across wider social networks, the relationship begins to organise everyday life for humans in practical and emotional ways—shaping routines, emotional pacing, and expectations of care in urban settings [3,20,21,22]. Rather than framing these dynamics in terms of harm or victimhood, this review approaches them as adaptive responses to broader social, technological, and relational conditions that have reshaped how intimacy, safety, and regulation are organised in contemporary life [8,23]. Seen in this wider context, the human–dog bond links canine welfare to the emotional organisation of modern societies [15,16,18], and shows how animals participate in shaping how regulation is lived in everyday urban environments [3,24,25].

This review focuses on Western, urban contexts, where companion dogs’ daily lives are shaped by dense regulatory, spatial, and moral infrastructures of care. The argument is not intended as a universal account of human–dog relations, but as an analysis of a specific socio-ecological configuration shaped by high regulation density, limited canine social continuity, and risk-averse caregiving norms.

## 2. Methods

This article is a narrative integrative review drawing on literature from canine behaviour science, comparative ethology, physiology, psychology, and the social sciences. The review focuses on how contemporary human–dog relationships are shaped by social fragmentation, urbanisation, and risk-avoidant caregiving, and how these relational patterns influence emotional regulation within and beyond the dyad. “Beyond the dyad” refers here to the organisation of everyday human environments, including routines, low-stakes sociability, and the conditions of contact in public space. Throughout, we use “guardian” to refer to the human caregiver of a companion dog, retaining “owner” only where it appears in legal terminology or in the wording of cited measures and datasets.

### 2.1. Search Strategy and Scope

Literature searches were conducted between October 2025 and January 2026. The primary focus was on peer-reviewed publications published between 2010 and 2025, a period marked by rapid growth in research on canine cognition, welfare, emotional regulation, and human–animal relationships. Earlier foundational works were included selectively where they provided essential theoretical frameworks, such as attachment theory, social buffering, affective neuroscience, and models of co-regulation.

Sources were retrieved using multiple databases and search tools to ensure broad interdisciplinary coverage, including Scopus, PubMed, and Google Scholar. Supplementary discovery tools (Consensus and Research Rabbit) were used for citation tracking and gap-filling rather than as standalone search engines. Specifically, they were applied to (i) identify highly cited or closely connected papers linked to key seed articles, (ii) follow forward/backward citations to locate relevant work outside the initial keyword strings, and (iii) detect recently published items that were not yet consistently indexed across databases. Their use was exploratory and iterative, and records identified through these tools were then evaluated against the same inclusion criteria as database-retrieved studies.

Search terms combined dog-related keywords with the main constructs reviewed here (co-regulation/synchrony, attachment/overprotection, social buffering/contagion, and urban social context).:

To increase transparency, we recorded retrieval counts for representative queries across databases (2010–2025). In Scopus (TITLE-ABS-KEY), concept-specific strings yielded 11 records for explicit co-regulation terminology, 105 for broader co-regulation/regulation/buffering terms, and 725 for contagion/synchrony/buffering terms; broader mapping queries returned 1693 (physiology/synchrony terms), 2260 (attachment/overprotection terms), and 3959 (urban/social context terms). In PubMed, targeted physiological queries returned 11 (synchrony/coupling with cortisol/oxytocin/HRV) and 7 (stress/emotional contagion with cortisol/oxytocin/HRV) results, whereas a broader attachment/overprotection query returned 962; a very broad HAI query returned 23,563 and was used only to indicate field scale. Google Scholar was used for additional field mapping and citation discovery; approximate result counts for title-only queries ranged from ~1880 to ~11,700 across key themes (synchrony/coupling; physiology; overprotection/pet parenting; social buffering), noting that Google Scholar provides dynamic, non-deduplicated estimates. Because the review was narrative and iterative (including citation tracking), these counts are reported as indicators of breadth rather than cumulative unique records.

### 2.2. Inclusion Criteria and Analytical Approach

Following title and abstract screening, publications were selected for full-text review based on their relevance to emotional regulation, social organisation, and relational dynamics in dogs, humans, or human–animal dyads. Eligible studies were peer-reviewed and either empirically examined behavioural, physiological, or social aspects of human–dog relationships, or provided theoretical models relevant to co-regulation, attachment, and social network structure.

Studies were excluded if they were anecdotal, lacked a clear methodological or conceptual framework, or focused narrowly on training techniques without addressing emotional, relational, or social dimensions. To contextualise canine findings within a broader evolutionary and social framework, a limited number of comparative studies on other social mammals (e.g., primates, rodents) and human-focused research were included. These references served as conceptual anchors rather than as primary empirical material.

The synthesis followed an integrative narrative approach, prioritising cross-study patterns, converging mechanisms, and conceptual linkages over exhaustive coverage or quantitative aggregation. Full texts were read iteratively and coded for recurring mechanisms and relational configurations, with themes refined by constant comparison across disciplines. To limit interpretive bias in a narrative review, thematic decisions and key integrative claims were refined through iterative co-author discussion and revision, rather than through independent duplicate screening. Searches and synthesis were conducted in English; non-English publications were not systematically targeted, but were included when identified and could be assessed using working translations.

### 2.3. Scope and Limitations

The final synthesis integrates empirical and theoretical literature across behavioural science, physiology, and the social sciences. As with much research on companion animals, the available literature is predominantly Western and urban in focus, with a strong emphasis on guardian-reported outcomes and dyadic human–dog relationships. Everyday social interactions among dogs outside guardian-mediated contexts remain comparatively underrepresented.

This also means that evidence on multi-dog households, cross-cultural caregiving norms, and developmental trajectories is comparatively thinner and uneven; where these areas are discussed, we treat them as boundary conditions that likely moderate dyadic dependence and regulatory outcomes rather than as fully mapped mechanisms.

These constraints influenced how the material was organised and interpreted. The analysis draws on converging work from several disciplines, but it is limited by what is currently available—especially the lack of data on multi-dog networks, longitudinal social development, and non-Western contexts.

## 3. The Architecture of Control in Contemporary Human–Dog Relationships

### 3.1. Overprotection and the Culture of Control

Contemporary human–dog relationships are embedded in dense systems of regulation that shape how care, safety, and responsibility are enacted in everyday life [26,27].

Contemporary cultures of care increasingly organise trust through precaution rather than through tolerance of uncertainty, embedding supervision and risk management into everyday relationships [2,8,28,29,30]. Law, city infrastructure, everyday routines, and moral ideas about good care all shape how dogs are expected to live—predictably, under supervision. This logic increasingly extends into everyday monitoring practices, where technologies such as GPS tracking or constant visual supervision are framed as care, while effectively redefining trust as continuous oversight [31]. This organisation works less through force than through concern: control is justified as protection, restraint as responsibility [31]. For humans, this shifts everyday regulation toward monitoring as a coping style [16]: calm becomes tied to predictability, surveillance, and avoidance of uncertainty [16,24], rather than to reciprocal adjustment [14,15]. To make sense of contemporary canine behaviour, these conditions need to be taken seriously.

Within these tightly managed social environments, dogs increasingly orient their behaviour and emotional regulation toward human guardians rather than canine partners. They look primarily to humans for social cues, replacing the subtle, reciprocal adjustments once exchanged with other dogs [32,33,34]. This substitution often feels safe, yet it fosters dependence. What was once a distributed network of regulation becomes concentrated within a single dyad, providing security while reducing flexibility [35,36]. Over time, stability is achieved more through predictability than through negotiated adjustment, leaving less room for repair, exploration, and autonomous recovery [31,37,38].

Most companion dogs today live as single pets [39,40,41]. Their social networks consist largely of episodic encounters with unfamiliar dogs, while the affiliative capacities evolved for long-term cooperation remain underused. This raises a fundamental question: what does social fulfilment mean for a species adapted to shared regulation and reciprocal care?

Importantly, this configuration is not universal even within Western societies. Multi-dog households can preserve more continuity in conspecific contact and may distribute regulatory work differently, potentially buffering dyadic overload—though the evidence is still patchy and often confounded by household routines and selection effects [7,42]. Cross-cultural caregiving norms and legal infrastructures also vary in how much uncertainty, proximity, and dog–dog contact they permit, which may shift the balance between control and negotiated adjustment [43,44]. Finally, developmental timing matters: early social experience and longitudinal trajectories are likely to determine whether human-oriented regulation becomes flexible competence or rigid dependence, yet these pathways remain under-tracked in urban samples [45,46,47].

Evidence from stable dog groups offers a contrast. In groups that remain together over time, dogs show steadier emotional states and fewer conflicts—a pattern observed both in domestic settings and in free-ranging populations [48]. At the same time, freer social choice in free-ranging contexts coexists with substantial physical and health risks, so “agency” here is not a welfare shortcut but part of a trade-off between autonomy and safety [48,49]. Here, “agency” refers to everyday behavioural options—everyday behavioural options—choosing distance, disengaging and returning on their own terms—that let dogs negotiate uncertainty—that let dogs negotiate uncertainty and adjust arousal through interaction rather than through restraint. The same competence—flexible cooperation and peaceful negotiation—persists in domestic dogs but often lies dormant in the managed solitude of urban life [49]. When those options are routinely reduced, stability can start to depend on limiting uncertainty rather than practising negotiation. A similar logic operates in urban human life, where safety is increasingly pursued through predictability and monitoring rather than through tolerance of uncertainty and negotiated adjustment.

Large-scale studies link these social patterns with canine emotional outcomes. Urban living, low activity levels, and limited early socialisation correlate with higher social fearfulness in dogs [1]. Ethnographic research adds a human dimension: guardians’ anxiety, social fatigue, or fear of conflict often limits dogs’ opportunities for contact—a pattern captured by the spillover hypothesis, where human stress and risk-avoidant coping reshape dogs’ social access through ordinary management decisions [50]. The pathway is often indirect: fewer repeatable, low-pressure encounters means fewer chances for familiarity, buffering, and social learning, which can stabilise fearfulness over time—especially in dogs already sensitised by early adversity [51,52]. At the same time, positive, structured human interaction can move fearful dogs toward safer expectations, so human influence can restrict contact, but it can also repair emotional trajectories [53,54]. In turn, restricted canine social life [55,56] can feed back into the household’s emotional climate, increasing vigilance [15] and reinforcing the very management practices meant to produce safety [18].

Although no quantitative model yet maps the full social networks of urban dogs [42,57], converging evidence suggests that most opportunities for dog–dog contact are shaped by human routines, rules, and space [41,43]. Dogs often grow into the social arrangements of their guardians. Much dog–dog contact is brief and episodic [57,58], often under conditions of human oversight, while continuity in dog–dog relationships remains poorly mapped [42].

This gap is amplified by the fact that multi-dog households, non-Western caregiving ecologies, and developmental change over time are rarely captured in the same datasets, making ‘the dog’s social world’ hard to represent as a network rather than a set of owner-mediated encounters [41,42,47,49].

The moralisation of safety has psychological consequences for both species and becomes embedded as a social norm. In everyday human life, autonomy increasingly unfolds under monitoring and precaution [29,31]. Uncertainty is handled as something to be reduced, not explored [31,38,59]. For dogs, it narrows the emotional space in which curiosity and regulation can unfold. Controlled environments interrupt the natural feedback loops of approach and retreat that support emotional resilience. Over time, both partners may learn a quiet confusion: calmness mistaken for immobility, stability for suppression [38].

In anthropological terms, it fits a broader shift in everyday trust relations. Contemporary societies increasingly reward emotional restraint—self-control, conflict avoidance, and affective neutrality [10,60]. In canine life, these values translate into behavioural inhibition, with the “well-behaved” dog serving as an emblem of emotional order [1]. Beneath that composure lies a shared tension: humans and dogs co-regulating not freedom, but constraint [9].

This culture of control does not arise from cynicism but from care [61]. It is rooted in fear—of harm, loss, and unpredictability—the same emotions that underlie attachment itself [11]. Efforts meant to protect can, over time, produce the same isolation they were supposed to avoid [23]. The paradox of modern guardianship is thus simple: affection sustained through control rather than connection [1,9]. In such settings, regulation shifts from a distributed, relational process toward a managed form of stability maintained through supervision and restraint.

### 3.2. Asymmetry, Infantilisation, and the Suspension of Canine Agency

Within this architecture of control, contemporary human–dog relationships display a growing asymmetry. Emotional closeness has intensified, while reciprocity has weakened. Dogs are more and more handled as socially fragile—assumed to need supervision at every step for their own good. Inside the human–dog relationship, this often means that the dog’s own capacity to choose, withdraw, or negotiate is put on hold.

The pattern is not unique to dog guardianship. In child supervision, good care is often defined as active oversight and risk prevention, adjusted to child age and environmental hazard, though risk-averse supervision can also narrow opportunities for exploration [62,63,64]. As dogs shift from functional roles to emotionally central family members and sources of psychosocial support, protection becomes easier to justify, and everyday choice—when to disengage, where to go, who to meet—shrinks accordingly [65,66,67], with consequences for how emotional safety is pursued in everyday life.

Psychological research on human–dog bonds indicates that high emotional investment does not necessarily produce relational balance; instead, certain attachment configurations are associated with heightened monitoring, control, and reliance on the dog as an emotional stabiliser [68,69]. For humans, this can shift emotion regulation toward supervision and predictability, with less room for reciprocal adjustment. Dog–dog interactions are typically sustained through negotiation and repair. In contrast, contact with humans is usually structured through guidance, rules, and management [36,42,48].

Behaviour is guided, corrected, or pre-empted rather than co-regulated. What is framed as care may therefore restrict opportunities for dogs to practise social skills, make decisions, or recover from uncertainty. Studies of attachment dynamics show that guardians with more anxious relational styles tend to engage in more controlling or intrusive caregiving, a pattern associated with increased distress and reduced behavioural flexibility in dogs [36,69]. Stability is achieved through oversight rather than shared regulation.

In this sense, agency is not a philosophical add-on but part of how regulation works. The ability to pause, take distance, opt out briefly—and return on one’s own terms—is one route through which arousal is adjusted in social systems [38,70]. When these micro-options are routinely removed, apparent stability can become a product of management rather than recovery [31,37]. The dyad may look calm, yet the dog’s regulatory repertoire narrows, leaving fewer everyday situations in which flexibility can be learned and expressed [1,32,35].

This asymmetry often emerges early [45,71]. Puppies raised primarily within human-centred environments learn to orient their regulation toward human cues rather than canine feedback [33,46]. While this orientation can enhance responsiveness to people, it may also limit opportunities to develop autonomous regulation and social problem-solving [32,35]. Research on dog–human attachment demonstrates that dogs’ behavioural outcomes are closely linked to caregiving style, with controlling or inconsistent interaction patterns associated with higher distress and poorer coping strategies [36,47,72,73].

With time, what might have developed as social competence is channelled into compliance, while room for autonomy gradually shrinks. Dogs remain emotionally close to humans yet become increasingly disconnected from the social processes that once distributed regulation across multiple partners.

The resulting pattern resembles what has been described as overattachment: a configuration in which emotional closeness intensifies while mutual regulation weakens [68,74]. In such relationships, dogs may function as compensatory attachment figures for humans with insecure relational histories rather than as socially mature partners [22,68,69].

Relatedly, household ecology (e.g., multi-dog living) and developmental history may moderate how strongly ‘care-as-oversight’ translates into suspended agency, but these moderators are rarely examined systematically [7,42,45,47].

This pattern does not signal neglect. It reflects a way of organising relationships in which protection and closeness gradually stabilise dependence. Comparative work suggests that this is not the only pathway observed in human–dog relationships. More task-oriented or functionally structured bonds, such as those in assistance dog contexts, are associated with lower emotional intrusion and greater behavioural independence [74].

## 4. Emotional Co-Regulation and the Mirror of Control

### 4.1. What Co-Regulation Is—And What It Is Not

Emotional co-regulation in human–dog relationships refers to a dynamic process of mutual adjustment [75], in which emotional states are shaped through ongoing interaction, responsiveness, and the possibility of recovery. What happens in co-regulation is not just emotional contagion.

While contagion describes the passive transmission of affect—such as stress or arousal spreading from one individual to another—co-regulation involves active modulation, flexibility, and the capacity to return to baseline through interaction [9,12,70,76,77]. Co-regulation depends on choice and responsiveness, but also on having alternatives—more than one way to regain balance. It does not stabilise emotion by holding behaviour still, but by making movement possible—approach and retreat, engagement and pause—without turning these shifts into a threat. In that sense, co-regulation preserves autonomy rather than overriding it.

Aligned patterns of arousal or affect can accompany both adaptive recovery and sustained vigilance, depending on the relational and environmental context in which they occur [9,23]. Emotional alignment does not guarantee regulation; it can equally stabilise tension. This distinction is especially relevant in human–dog relationships, where emotional closeness is often taken as a given good. The presence of emotional synchrony or perceived closeness alone does not indicate healthy regulation [78,79]. The question is whether synchrony can shift with context, or whether it settles into a rigid, repetitive pattern held in place by restraint.

### 4.2. From Distributed Regulation to Dyadic Dependence

Dogs are not emotional regulators by definition. Rather, they have become embedded within relational systems in which emotional regulation increasingly concentrates within the human–dog dyad. As broader social networks contract, functions once distributed across multiple, predictable relationships are absorbed by the dog, transforming co-regulation into a compensatory mechanism [6,9,11,27,80]. This concentration of regulatory functions within a single relationship mirrors broader zero-risk orientations, where efforts to eliminate uncertainty paradoxically reduce systemic resilience [27]. By “zero-risk orientations” we mean a cultural and institutional preference for eliminating adverse outcomes in advance, treating uncertainty as something to be removed rather than held and negotiated [81,82].

When social options narrow, emotional balance stops circulating and settles into pairs. What follows is not isolation, but a transformation in how regulation itself is organised. Attachment becomes concentrated in one direction: toward the human guardian. The dog’s calibration of safety increasingly depends on a single regulatory figure, while the human, in turn, draws reassurance from the dog’s compliance.

This concentration is likely to be strongest in single-dog, single-guardian arrangements. In multi-dog households, regulatory dynamics may be distributed across more than one relationship, and stable conspecific contact can provide an additional buffering route that reduces exclusive reliance on the human partner [42,48]. This does not remove dependence risk, but it can change its geometry—from a single dyadic corridor to a more networked form of regulation [49].

For humans, this can externalise regulation into everyday management—calm becomes tied to monitoring, routines, and predictability [16,18]—shaping not only the relationship but the person’s tolerance of uncertainty [16] and patterns of social engagement [3,24].

This configuration produces asymmetric co-regulation. Emotional equilibrium is maintained not through mutual recovery but through containment [37]. Predictability replaces negotiation; stability is achieved by limiting variability rather than by tolerating it. Similar dynamics have been described as “surveillance as care,” where monitoring and restriction are negotiated as responsibility and concern rather than mistrust [31]. For dogs, containment is often maintained by shrinking everyday room for self-directed adjustment—so regulation becomes less about flexible recovery and more about staying within a narrow, managed band of safety [31,37,83].

Attachment research helps clarify this shift. Secure relational patterns are associated with co-regulation that supports exploration, autonomy, and flexible recovery, whereas anxious or overinvolved configurations tend to reorganise regulation around monitoring, intrusion, and control [69].

Urban rules and spaces make this concentration even stronger. Dogs increasingly become positioned as emotional stabilisers for humans [3,11], while their own regulatory repertoires narrow [1]. They inherit human stress but lack access to the peer interactions that once dispersed it. In this sense, the human–dog dyad can function as an emotionally fortified micro-space, echoing broader strategies of urban risk containment and defensive living [84].

This shift does not reflect a failure of care. It reflects a relational system in which dependence is maintained through protection and proximity, and where emotional work is concentrated within a single, unequal relationship. What diminishes is not attachment itself, but the distributed conditions that once made regulation resilient.

**Figure 1:**
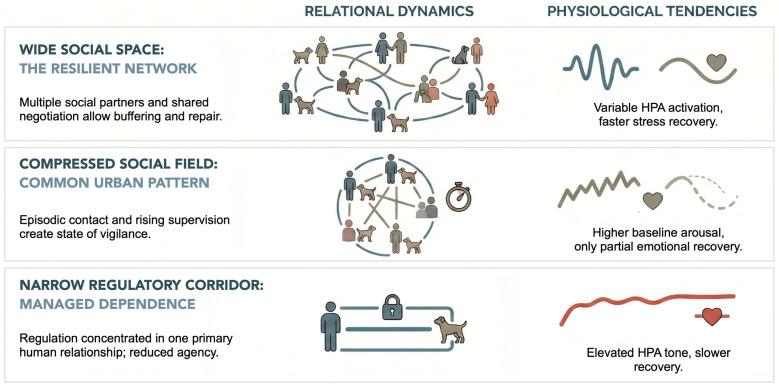
Conceptual schematic of contraction in social regulatory space and its relational and physiological correlates. Three analytic configurations are shown: wide social space, compressed social field, and narrow regulatory corridor. The relational column describes the organisation of regulation; the physiological column lists expected correlates (HPA tone/recovery) as tendencies discussed in the reviewed literature.

To visualise this shift, Figure 1 summarises three ideal-typical configurations of dogs’ social environments: a wide social space (multiple regulators and repeatable, negotiable contact), a compressed social field (contact without continuity, often under supervision and with limited repair), and a narrow regulatory corridor (regulation concentrated in the human–dog dyad and stabilised through predictability and restriction). The relational column indicates how regulation is organised in each configuration; the physiological column lists expected correlates (e.g., HPA tone and recovery) discussed across the reviewed literature. The tiers are analytic simplifications rather than discrete categories or diagnostic states.

### 4.3. Physiological Coupling as a Pathway, Not a Cause

Physiological coupling may act as one biological route through which relational dynamics are maintained over time [15,85]. This matters because coupling can carry emotional states across the dyad [15,18], shaping human affective baseline and the felt stability of everyday life [16,17]. Studies of interspecies synchrony show that dogs often mirror human physiological and hormonal states, including heart rate, cortisol, and oxytocin [12,86]. Importantly, these measures are not emotion-specific: similar profiles can accompany different affective states, including affiliative arousal, vigilance, or distress [12,13]. In managed contexts, “calm” can become the explicit goal, yet reduced outward arousal may reflect containment or inhibition rather than recovery [38,51].

Dogs seem finely tuned to human signals—a sensitivity forged over long co-evolution in shared emotional settings [13,87]. Hormonal synchrony is easiest to spot under acute stress, though it can also take shape gradually, in less obvious ways. Hair cortisol studies report correlated levels between guardians and dogs across seasons, consistent with sustained interspecies stress mirroring [12]. Brief affiliative moments—especially touch and play—are linked to oxytocin release in both dogs and humans, helping interactions settle and open up, at least in the short term [86,88].

However, bonding chemistry does not inherently signal healthy regulation. Importantly, canine studies indicate that oxytocin release does not reliably signal emotional relaxation or secure regulation. In dogs, oxytocin can be released alongside elevated cortisol during socially salient or stressful interactions, suggesting a role in coping and proximity-seeking rather than simple stress reduction [13,78,79,89]. These findings challenge interpretations of oxytocin as an unambiguous marker of positive welfare and support the view that bonding-related neurochemistry may also stabilise vigilance under conditions of uncertainty. Sustained oxytocin co-activation under conditions of limited autonomy or chronic stress may reinforce proximity-seeking and dependence rather than resilience [12,61]. Research on overparenting suggests that such stabilisation may reflect caregivers’ own attachment needs and affect-regulation strategies, rather than the developmental needs of the dependent partner [61,90]. Physiological work further suggests that caregivers’ capacity for co-regulation is constrained by their own autonomic regulatory flexibility, indexed for example by respiratory sinus arrhythmia (RSA); lower regulatory capacity may therefore bias dyadic regulation toward control and stabilisation rather than shared recovery [38]. In such contexts, physiology records social design: biological systems adapt to relational constraints rather than correcting them.

A similar view is taken in Social Safety Theory. Social Safety Theory frames stress physiology as primarily calibrated by cues of social safety versus social threat (e.g., belonging, predictability, conflict), rather than by objective stressors alone [83,91]. Here, stress-related physiology is understood as reacting mainly to signals of social safety or threat, not to single stressors in isolation. Prolonged relational uncertainty, in this sense, can sustain defensive biological states even in relatively benign conditions [92].

Evidence from human mental health research suggests that similar dynamics may carry costs for humans as well. Stronger attachment to companion animals is not consistently associated with better psychological well-being [93]. In certain high-risk contexts, however, dogs may play emotionally protective role, including a lower risk of suicide [94]. At the same time, longer-term data point in a different direction. Stronger emotional attachment to dogs has been associated with declines in psychological well-being [93]. Other work suggests that anxious or overinvolved forms of pet attachment are linked to poorer mental health, especially when relationships are marked by behavioural problems, or a sense of mismatch [22,95].

When dogs live in socially impoverished or unstable settings, their HPA-axis activity shifts accordingly. Baseline cortisol tends to rise, recovery after stress takes longer, and arousal is less easily down-regulated [47,96,97]. Similar profiles have been reported in humans and primates when attachment cues are unreliable or inconsistent [45]. In dogs, the lack of canine partners shifts more of the regulatory load onto humans, making dyads more susceptible to stress spillover and mutual vigilance [7,12]. Under these conditions, the dog may be less able to buffer human stress [16,18], and synchrony may tilt toward shared vigilance rather than recovery [12,15].

This has a direct welfare implication. If a dog is expected to function as a primary stabiliser within a constrained dyad, the cost may be reduced behavioural flexibility [7,69]. In this review, behavioural flexibility is treated as an output of the regulatory system: the capacity to explore, to regain baseline after stress, and to tolerate uncertainty without escalating into avoidance or inhibition [47,92]. When the dog’s own social buffering is limited and regulation becomes organised around predictability and monitoring, these outputs can narrow—even when the dyad appears “calm” [1,31]. Over time, what looks like stability may reflect management-compatible inhibition rather than resilient recovery [13,38].

Physiological coupling does not generate these dynamics; it stabilises them. Over time, zero-risk orientations may therefore increase physiological load rather than reduce it, trading short-term predictability for long-term loss of regulatory flexibility [28]. When human emotional environments are shaped by vigilance or anxiety, synchrony can sometimes serve to pass these states along within the dyad, rather than buffering them. Calm may emerge, but it is a calm sustained by control rather than by recovery. Apparent calm may therefore reflect inhibited responding rather than recovery: reduced overt arousal can coexist with a defensive physiological state and limited autonomic flexibility, rather than relaxed social engagement [38].

What appears as emotional balance increasingly reflects containment—an equilibrium maintained through predictability and restraint rather than through shared adjustment. These asymmetrical loops form the emotional foundation of what follows: the biology of containment disguised as care.

Table 1 summarises this contrast by treating adaptive co-regulation versus maladaptive dependence as relational patterns and by linking “recovery” versus “containment” to expected profiles in cortisol, HRV, oxytocin-related processes, and everyday function.

## 5. Dogs as Anchors of Embodied Social Life

Here we focus on how dogs and dog–human coordination reshape human rhythms, attention, and low-stakes sociability in public space.

Dogs do not only adapt to urban life; they can also reshape its everyday rhythms. Through repeated walking routes, pauses, and brief encounters in shared space, they structure when and where people move, who they end up speaking to, and how ordinary public contact becomes possible or avoided [3,98]. This anchoring role is especially visible in risk-averse, digitally mediated settings, where contact may be frequent yet less physically shared and less available for everyday co-regulation.

This is not a collapse of care but a shift in its social function: control is recast as protection, management as love, and regulation as safety [99,100]. Within this framework, dogs mirror their time—emotionally attuned to humans yet shaped by norms of predictability and oversight [15,24]. At the same time, dogs exert a countervailing influence on human life: even within tightly managed environments, they draw people back into the physical world in ways that resist full digital mediation [3,17]. This embodied engagement supports mental health and social connection in ways that purely digital interactions cannot fully replicate [16,101].

This “return” happens through rhythm and constraint. Dogs require movement, repeated outdoor exposure, and recurrent scheduling, thereby creating everyday opportunities for contact that are difficult to fully optimise or virtualise [19]. Crucially, this is not only about being outside; it is about reintroducing analog friction—the tactile and physical interactions often missing in digital life: weather, uneven ground, waiting, stopping, detours, and bodily effort. In work contexts, especially during teleworking, companion dogs have been shown to attenuate the impact of daily hassles and reduce uncertainty through direct, real-world companionship [16]. In regulatory terms, friction matters because it supports closure of arousal cycles through action and sensory feedback, rather than leaving stress to circulate as abstract, screen-based vigilance.

Recent work also highlights the limits of mediated substitutes for this embodied co-presence. While emerging technologies attempt to support remote human–dog interaction, available evidence suggests these mediated alternatives remain less effective than direct physical contact in supporting mental health and social connection [17,101]. This reinforces a simple point: if the dog functions as an anchor at all, the anchoring is not informational; it is bodily.

This anchoring role, however, is not uniformly restorative. As established earlier (see Section 3.2), it can also operate as a compensatory emotional strategy when dogs become primary figures of comfort in fragmented social lives. In such cases, stronger attachment to dogs is not consistently associated with better well-being and may correlate with poorer psychological outcomes, particularly when it compensates for insecure or strained human attachment [69]. This does not imply that preferring interspecies companionship is pathological. The concern arises when this preference reflects constrained options or reduced social choice—so that regulation becomes concentrated by necessity rather than by freely chosen relational ecology. The concern is not attachment itself, but concentration of regulatory burden within the dyad, where daily affect regulation and tolerance of uncertainty may become organised around a single relationship. Even so, beyond the private dyad, dogs also shape the public conditions under which low-stakes contact becomes possible.

In the public sphere, dogs operate as participants in embodied forms of social exchange. Observational studies consistently show that the presence of a dog lowers the threshold for approach between unfamiliar humans, reducing the interpersonal cost of contact and making brief interaction socially permissible [3]. These encounters are rarely planned: they occur through pauses, small exchanges, distance negotiations, and shared attention to movement in public space. The mechanism is partly spatial and bodily: people adjust pace and trajectory in response to dogs, coordinating movement through micro-acts of negotiation.

At the micro-level, this often takes the form of behavioural synchrony—shared pacing, stopping, and scanning [14,102,103]. This behavioural alignment may be accompanied by interspecific neural coupling during mutual gaze and touch, consistent with shared attentional states within the dyad [15]. In practice, humans often structure the immediate context (route, pace, pauses), while dogs actively monitor and adjust to subtle cues to maintain proximity and social cohesion [85].

Beyond single encounters, repeated visibility in shared spaces supports recognisability and the formation of weak but persistent ties—relationships that remain low-intensity yet can accumulate into informal support and neighbourhood familiarity [55,104]. Over time, dog walking can thus generate public familiarity: public space becomes more emotionally legible through recurrence—faces, timings, routes, and small scripts of acknowledgement. Dog guardians can also contribute to “eyes on the street”: regular presence becomes a low-level, distributed form of neighbourhood awareness that supports routine sociability in shared space [24,25]. Importantly, this does not require deep relationships; it operates through repeated exposure, recognisability, and low-stakes interaction.

Taken together, dogs function as informal infrastructures of embodied sociability. They reintroduce movement, analog friction, and repeated public familiarity into settings organised around efficiency and avoidance [3,98]. This can strengthen human social health, but it is conditional—shaped by local norms, perceived risk, and the dog’s social affordances [25]. The same anchoring that benefits humans can still carry costs for dogs when it is sustained through asymmetrical dependence rather than reciprocal social buffering [69,85]. In other words, dogs reshape human social experience and the texture of public life—even as they remain constrained by relational conditions not of their own making.

## 6. Ethical and Welfare Considerations

Ethical frameworks that treat the human–dog bond mainly as an end in itself can miss what accumulates over time in everyday life with dogs [100]. Relational closeness is often taken for granted as protective, yet this assumption leaves little room to notice forms of overload—constant availability, heightened vigilance, or the pressure to regulate human emotion [7,15,18,22]. From a welfare perspective, the issue is therefore not only how dogs are cared for, but where regulatory responsibility is placed, and how narrowly it becomes concentrated within the human–dog dyad [18,27,29]. From the human side, this can normalise coping through supervision and predictability [16,18], reshaping everyday emotional environments while increasing the dog’s regulatory load [15].

Ethically, this argues for expanding everyday freedoms for both partners: more canine choice (distance, exploration, repeatable dog–dog contact) and less human reliance on supervision as the default route to feeling safe.

If dogs function as elements of everyday emotional infrastructure, welfare costs cannot be treated as incidental. Regulatory load is not only about stress exposure; it is also about what the dog may trade away to keep the dyad stable—behavioural flexibility, exploration, and the option to disengage [20,35,37]. In this sense, agency is not a philosophical add-on but part of how regulation works: the ability to pause, choose distance, withdraw, and re-enter interaction on the dog’s own terms is one of the mechanisms through which arousal is adjusted in social systems [38,70]. Stable conspecific social buffering matters in the same structural way. When repeatable dog–dog relationships are scarce, more regulation is forced into the human–dog corridor, increasing the likelihood that synchrony stabilises restraint rather than supports recovery [12,13,48]. Ethical evaluation should therefore include not only the quality of human care, but the dog’s access to choice and to social continuity beyond guardian-managed encounters [99,100]—one reason idealised narratives can obscure where regulation becomes a demand.

Talking about dogs in terms of “unconditional love” affects how these relationships are understood. They suggest that the bond is natural, limitless, and free of cost. Framed this way, care appears simple and endless—which makes its costs easy to miss. In this view, a dog’s commitment is treated as something—part of what dogs are—rather than something that develops through interaction and depends on conditions. The possibility that a dog might need distance, withdrawal, or relief from emotional demands tends to disappear from the picture. Dependence does not disappear; it becomes familiar and therefore less visible. Questioning unconditionality does not mean questioning attachment. It means recognising that care always has a structure. When that structure is hidden behind idealised language, it becomes harder to notice where responsibility accumulates and how limited a dog’s room for choice may be.

Research on human–dog relationships has increasingly focused on attachment and emotional regulation. These approaches offer important insight into interspecies dynamics. At the same time, they bring ethical questions of their own—about proximity, responsibility, and the emotional demands placed on dogs—that deserve to be addressed directly.

Work on emotional synchrony or stress responses risks shaping the processes it tries to capture, even when disruption is unintended. This reactivity can apply to both partners: dogs may respond to unfamiliar equipment, handling, or altered routines, which can shift arousal and interaction patterns independent of the relationship itself [31,92]. Human awareness of being studied, particularly in emotionally sensitive contexts, can alter caregiving behaviour, levels of control, or expressions of concern, thereby reshaping the relational environment of the dog [31]. Ethical research design therefore calls for limiting intrusion as much as possible and favouring observation that stays close to everyday conditions. Where physiological sampling is necessary, protocols should minimise novelty and handling effects (habituation to equipment, low-interference recording, and designs that preserve ordinary routines), because measurement itself can shift arousal and interaction patterns.

Physiological measures, including cortisol sampling or heart-rate monitoring, should be interpreted with caution and employed only when their potential welfare costs are outweighed by clear scientific value [92]. Repeated handling or unfamiliar experimental setups can add stress of their own, especially for dogs already living in a state of heightened vigilance or with little access to social buffering.

An additional ethical challenge concerns interpretation. Framing behavioural or physiological patterns as indicators of dysfunction risks pathologising adaptive responses to constrained social environments [28]. In zero-risk oriented cultures, such adaptations may stabilise behaviour in the short term while gradually eroding relational resilience for both dogs and humans over time. Researchers must therefore remain attentive to contextual factors and avoid conclusions that could reinforce excessive control, surveillance, or restrictive management practices in everyday guardianship.

Finally, research in this field carries broader social implications. Findings on emotional dependence or synchrony rarely stay within academic debate. They shape public attitudes toward dogs and, over time, filter into training practices and policy decisions [61,84].

Ethical responsibility thus extends beyond data collection to the ways in which results are communicated, ensuring that scientific insights support welfare without legitimising further social reduction or loss of agency for either species [99,100].

## 7. Conclusions and Future Directions

Dogs, by their evolutionary history, function as social regulators [34,105]. That is, they modulate arousal and behaviour through ongoing, reciprocal adjustment within relationships—by tracking others, negotiating distance, and repairing tension over time. Emotional balance relies on predictable relationships and the chance to adjust to others over time. In many cities, such relational structures are now fragmented. What once operated as a loose, multispecies system of co-regulation has increasingly narrowed into managed pairs: a human and a dog, each maintaining the other within limited corridors of safety [9]. This shift extends beyond behaviour to the organisation of social life itself. A species shaped for social intelligence now inhabits routines that prioritise quiet over negotiation and compliance over shared regulation.

Across urban contexts—both legal and emotional—a parallel pattern emerges in which trust is less often enacted through relational tolerance of uncertainty and more often stabilised through supervision, precaution, and control. In practice, dogs are left with limited scope to let relationships emerge, stabilise, or recover after tension. For humans, it fosters strategies of safety grounded in supervision rather than reciprocity [2,8,9]. The resulting state is one of mutual watchfulness, where safety is maintained by managing and limiting what happens next. Calm is sustained through restraint rather than recovery. What diminishes is not attachment itself, but co-regulation: the dynamic exchange through which safety is distributed and emotion stabilised.

For humans, this reorganisation can shape not only emotional states, but also everyday movement [103,106], social contact [3,104], and the felt openness of public space [24,25]—pathways through which dog–human coordination may matter for physical and social health [7,104].

This configuration reflects a broader cultural condition. The same sensitivity to risk and unpredictability that structures contemporary human life also shapes the management of dogs [2,6,8,107]. Instruments of control—leashes, fences, behavioural rules, and regulatory frameworks—extend a collective nervous system increasingly oriented toward vigilance. Through close emotional ties, dogs take on these patterns of restraint, becoming part of a shared, carefully managed calm [108]. In this sense, changes in canine sociability do not stand apart from human experience, but index a wider contraction in how social trust and emotional openness are organised.

Reframing behaviour and welfare within this context alters their interpretation. What is often described as reactivity, fear, or dependence may be better understood as an adaptation to limited social worlds rather than as individual pathology [1]. They arise in settings where social continuity and mutual regulation are reduced. From this perspective, emotional and behavioural outcomes in dogs offer insight into the relational conditions under which regulation is sustained or eroded in social systems more broadly.

The implications extend beyond companion animals. As a species shaped by co-regulation, dogs make visible a principle that also governs human emotional life: stability is not achieved in isolation, but emerges through reliable, reciprocal relations. Because dogs’ everyday routines and access to social contact are tightly constrained by human management and urban rules, changes in their social worlds can act as a marker of broader constraints in human social organisation.

Looking at these dynamics makes it hard to separate canine welfare from human social life. Paying attention to how dogs cope with urban social life also sheds light on how humans structure their own. The same arrangements that shape canine regulation shape human relationships as well. Seen this way, dogs can index wider social conditions rather than standing as isolated welfare cases.

This points to two priorities for future work: (i) mapping dogs’ everyday social worlds beyond guardian-managed encounters, and (ii) testing longitudinally whether early conspecific continuity predicts later regulatory flexibility—i.e., when dyadic synchrony supports recovery versus when it stabilises vigilance under control-heavy caregiving. These questions likely depend on household ecology (e.g., multi-dog living) and on non-Western or rural caregiving arrangements that redistribute regulatory load across more partners and routines.

The review also suggests practical implications for urban environments. If regulation is maintained through narrowed options, welfare and social functioning improve not only through “more calm” but through more negotiable space—conditions that allow approach–retreat cycles, disengagement, and low-pressure re-engagement. For dogs, this means enabling repeatable, low-arousal contact where relationships can stabilise (not only brief, high-intensity encounters), and designing routines that preserve exploration and choice alongside safety. For humans, it means relying less on supervision as the default route to feeling safe, and more on predictable, low-stakes sociality that does not require constant monitoring. The aim is not to eliminate management, but to shift from control that suppresses uncertainty toward the cultivation of low-stakes micro-communities and repeatable social routines that can hold it—so that synchrony, when it occurs, is more likely to function as buffering rather than as containment.

Together, these directions reinforce the core argument of this review: emotional regulation in human–dog relationships cannot be understood in isolation from the social environments in which both species live.

## Figures and Tables

**Table 1:** Contrasts adaptive co-regulation with maladaptive dependence not as individual traits, but as relational patterns shaped by broader emotional and social contexts.

Dimension	Adaptive Co-Regulation	Maladaptive Dependence
HPA Axis (Cortisol)	Flexible cortisol variability; stable circadian rhythm; short recovery after stress	Flattened variability or chronically elevated baseline; prolonged recovery
Autonomic System (HRV)	HRV synchrony during positive interactions; independence during challenges	Excessive HRV coupling across all contexts; reduced self-regulation
Oxytocin and Bonding	Oxytocin peaks during affiliative contact (gaze, touch); supports recovery	Sustained oxytocin co-activation across contexts, maintaining dependence and stress contagion
Guardian Profile	Secure attachment; low chronic stress; balanced responsiveness within everyday social contexts	Anxious or avoidant attachment; high neuroticism; chronic vigilance embedded in daily routines
Canine Behaviour	Balance between proximity and exploration; rapid return to baseline	Excessive proximity-seeking; reduced exploration; problem-solving deficits
Functional Outcomes	Stress buffering; social flexibility; adaptive learning across relational settings	“Managed calm”; reduced flexibility; inhibition as stability

## Data Availability

No new data were created or analyzed in this study. Data sharing is not applicable to this article.
